# The Effect of Temper Condition and Feeding Speed on the Additive Manufacturing of AA2011 Parts Using Friction Stir Deposition

**DOI:** 10.3390/ma14216396

**Published:** 2021-10-25

**Authors:** Mohamed M. Z. Ahmed, Mohamed M. El-Sayed Seleman, Ebtessam Elfishawy, Bandar Alzahrani, Kamel Touileb, Mohamed I. A. Habba

**Affiliations:** 1 Mechanical Engineering Department, College of Engineering at Al-Kharj, Prince Sattam Bin Abdulaziz University, Al Kharj 16273, Saudi Arabia; ba.alzahrani@psau.edu.sa (B.A.); k.touileb@psau.edu.sa (K.T.); 2 Department of Metallurgical and Materials Engineering, Faculty of Petroleum and Mining Engineering, Suez University, Suez 43512, Egypt; mohamed.elnagar@suezuniv.edu.eg (M.M.E.-S.S.); ebtessam.elfishawy@aucegypt.edu (E.E.); 3 Mechanical Engineering Department, School of Science and Engineering, The American University in Cairo, 11835 New Cairo, Egypt; 4 Mechanical Department, Faculty of Technology & Education, Suez University, Suez 43518, Egypt; Mohamed.Atia@suezuniv.edu.eg

**Keywords:** friction stir deposition, solid-state additive manufacturing, AA2011-T6 and AA2011-O, AA2011 aluminum alloy, microstructure, intermetallics, hardness

## Abstract

In the current study, solid-state additive manufacturing (SSAM) of two temper conditions AA2011 was successfully conducted using the friction stir deposition (FSD) process. The AA2011-T6 and AA2011-O consumable bars of 20 mm diameter were used as a feeding material against AA5083 substrate. The effect of the rotation rate and feeding speed of the consumable bars on the macrostructure, microstructure, and hardness of the friction stir deposited (FSD) materials were examined. The AA2011-T6 bars were deposited at a constant rotation rate of 1200 rpm and different feeding speeds of 3, 6, and 9 mm/min, whereas the AA2011-O bars were deposited at a constant rotation rate of 200 mm/min and varied feeding speeds of 1, 2, and 3 mm/min. The obtained microstructure was investigated using an optical microscope and scanning electron microscope equipped with EDS analysis to evaluate microstructural features. Hardness was also assessed as average values and maps. The results showed that this new technique succeeded in producing sound additive manufactured parts at all the applied processing parameters. The microstructures of the additive manufactured parts showed equiaxed refined grains compared to the coarse grain of the starting materials. The detected intermetallics in AA2011 alloy are mainly Al_2_Cu and Al_7_Cu_2_Fe. The improvement in hardness of AA2011-O AMPs reached 163% of the starting material hardness at the applied feeding speed of 1 mm/min. The hardness mapping analysis reveals a homogeneous hardness profile along the building direction. Finally, it can be said that the temper conditions of the starting AA2011 materials govern the selection of the processing parameters in terms of rotation rate and feeding speed and affects the properties of the produced additive manufactured parts in terms of hardness and microstructural features.

## 1. Introduction

Additive manufacturing (AM) is a promising technology in numerous engineering applications. It involves the fabrication of various 3D objects by adding layer by layer material (alloy, plastic, concrete, human tissue, etc.) regardless of any size and shape [1,2]. Fusion-based additive manufacturing (F-BAM) techniques are used for different alloys [3]. Still, they are not suitable for aluminum-based alloys, especially the heat-treatable alloys (2xxx and 7xxx), due to their sensitivity to porosity formation, liquation cracking, segregation, solidification cracking, and anisotropic microstructure [2,4]. In contrast, friction stir deposition (FSD) is solid-state additive manufacturing (S-SAM) technique that can be used to deposit metals and composites [5,6]. The main advantage of the FSD as a solid-state process is that it can eliminate all problems of melting and solidification, and also, the feed material is mainly rods or wire without a need for special specifications of the used feed material. Most of the current works today are carried out on AM of aluminum-based alloys using a S-SAM technique [1,7,8]. In recent years, there has been increasing interest in utilizing FSD in many applications. This technique can be used for many purposes, including additive manufacturing [1,9,10,11], surface protection [11,12,13], and repair of defective components [6]. Thus, it can be said that the FSD-based AM is considered a new innovative approach to AM for building 3D parts ultimately in a solid state. The main processing parameters are the rotation rate, feeding speed, downward force, consumable rod material, and substrate material. These parameters govern the heat input and material flow processes. In the FSD process, the final build part’s height depends on the layer thickness and the total number of assembly layers. Moreover, the modifications in the geometry of the building design can obtain manufacturing parts with different geometries [1,5]. Thus, the final FSD product is a near-net-shape with enhanced microstructure and isotropic mechanical properties [2,4,14]. Low porosity and low residual stress are the main privileges of the as-deposited part; this will make post-processing heat treatment unnecessary in many cases. However, surface finishing will usually be required [15,16,17]. Boeing and Airbus companies are considered the first use of additive manufacturing (AM) based on FSW principles [10,18,19]. Meanwhile, Airbus [20] presented the capability of achieving lightweight/low-cost structure parts by manufacturing 2050 Al-Li wing ribs by the FSAM process. Boeing assessed this process as a pre-form fabricating tool for manufacturing energy-efficient structures [10,21,22]. In addition to the capability of the FSD technique to generate material builds of high-performance structures, it is considered an energy and cost-saving process [10,23]. Elfishawy et al. [24] studied the possibility of multi-layers formation of die-cast Al–Si via FSD at the spindle rotation rate of 1200 rpm and different feeding speeds from 3 to 5 mm/min. The results showed sound structure with recrystallized refined grains. Therefore, from a scientific and technological point of view, it is of great importance to study how FSD works for additive manufacturing parts (AMPs) production in heat-treatable aluminum alloys. Although AA2011 is used extensively in aerospace and automotive components, there is a lack of publications discussing the applicability of AA2011 fabrication using FSD. Thus, the current work intends to explore the effect of the initial material conditions of AA2011 alloys on the properties and microstructures of the final produced AMPs. Three levels of feeding speeds of 3, 6, and 9 mm/min were associated with a high rotation rate of 1200 rpm/min to friction stir deposit AA2011-T6, and three other feeding speeds of 1, 2, and 3 mm/min were chosen with a low rotation rate of 200 rpm/min to deposit AA2011-O.

This study aims to study the effect of the consumable rod alloy temper condition on the behavior of the FSD process in terms of the parameters suitable for each temper condition as well as the properties of the AMPs.

## 2. Materials and Methods

**Figure 1:**
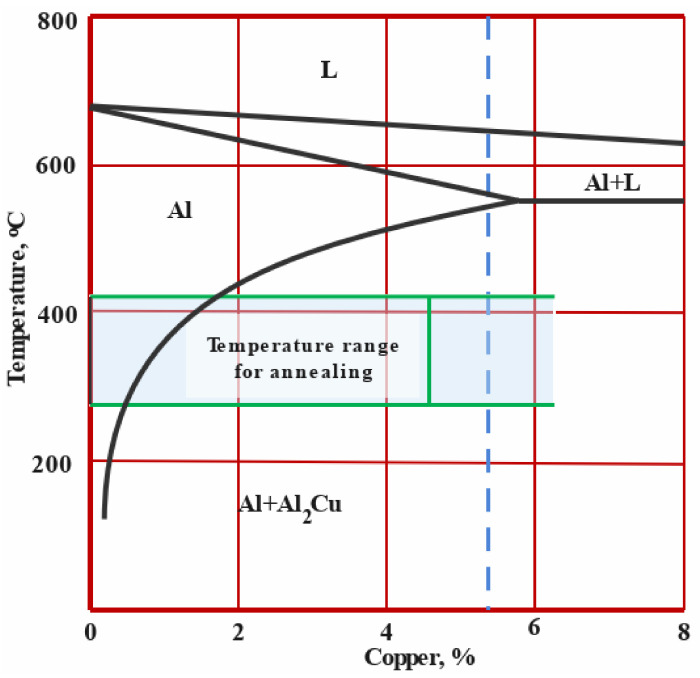
A sketch for the Cu-rich portion of the Al–Cu binary phase diagram with the annealing temperature range indicated.

To study the effect of the temper condition of AA2011 alloy on the properties of the produced AMPs, two groups of specimens, AA2011-T6 and AA2011-O, were used as consumable bars against a substrate of AA5083 alloy. The nominal chemical composition of AA2011 is given in Table 1. The annealing process for the as-received was carried out at 415 °C for 2.5 h followed by slow furnace cooling to the room temperature. Figure 1 illustrates the Cu-rich portion of the Al–Cu binary phase diagram with the annealing temperature range indicated [25,26].

For comparison, three deposited materials were manufactured from each group of the AA2011-T6 and AA2011-O rods. The FSAM was carried out using the friction stir welding/processing machine (EG-FSW-M1) (Suez University, Suez, Egypt) [27,28]. Table 2 summarizes the deposition process parameters of both Al alloys.

**Figure 2:**
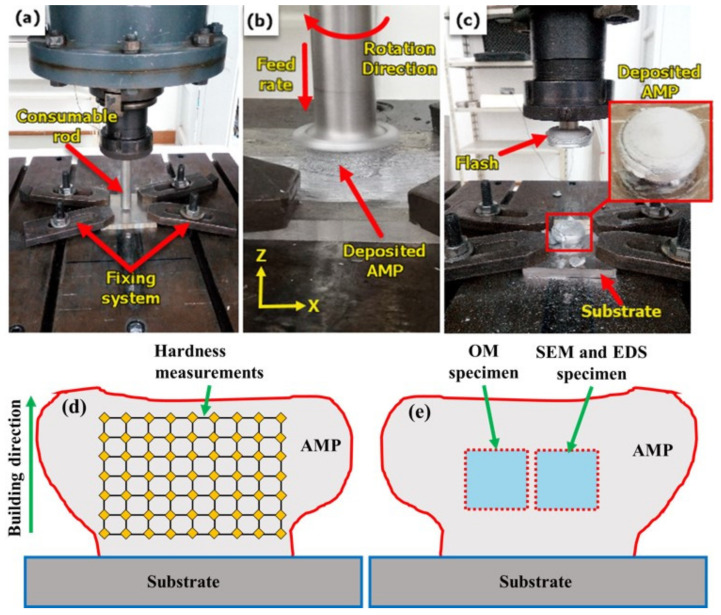
Photographs for the stages of the FSD process: (**a**) Fixing the AA2011 consumable rod and substrate AA5083 on the FSW/FSP machine, (**b**) feeding process during the FSD showing the building up of the part, and (**c**) the end of the deposition process for the additive manufacturing part (AMP). (**d**,**e**) are schematic drawings of the AMP sections showing hardness measurement points, and the second half of AMP shows the specimens cut for OM and SEM examinations, respectively.

The consumable aluminum rods are fixed using the machine shank to ensure the complete fixation of the rods throughout the process; Figure 2 shows a photograph of the actual AM process applied to AA2011. The additive manufacturing (AM) process involves three steps: fixing the consumable Al rod in the spindle shank (Figure 2a) and rotating it at a constant rotation rate while moving downward to reach the substrate material (Figure 2b). Finally, under a continuous feeding speed, the rod plastically deformed due to the high friction and the generated heat between the rod and the substrate that causes the material to transfer from the consumable bar to the substrate to build a material upwards. This process may continue until all the rod length is consumed and became insufficient for more deposition. The shape of the consumed tool tends to form a conical shape, as shown in Figure 2c. For AA2011-T6 group specimens, careful processing parameters were selected based on our experience in the field and the published data [24,29] to produce additive manufacturing parts. The required heat input to friction stir deposit such a hard material limits the process parameters to be 1200 rpm as a spindle rotation rate with 3, 6, and 9 mm/min feeding speeds. For the AA2011-O group specimens, experiments start with shortening each of the three specimens to 110 mm in length. Of this length, 70 mm of the total length was consumed as a fixing base of the rod inside the shank to ensure tight gripping and prevent rod deflection during the deposition process, and 40 mm was functional during the process of friction deposition. Less heat input is needed to deposit this soft material; that is why after many trials, the optimum process parameters obtained were a 200 rpm spindle rotation rate and feeding speeds of 1, 2, and 3 mm/min. Figure 2d,e show schematic drawings of the AMP sections showing hardness measurement points, and the second half of AMP shows the specimens cut for OM and SEM examinations, respectively.

Additive manufacturing parts (AMPs) have been sectioned vertically along the building direction (z-direction). The deposited layers were oriented perpendicular to the specimen axis/loading direction. The longitudinal sections were prepared according to the standard metallographic procedures by grinding up to 0.05 μm alumina polishing surface finish. The polished sections were investigated using an optical microscope (Olympus, BX41M-LED, Tokyo, Japan) after etching according to ATSM standard E407 using Keller’s etchant of the chemical composition of 100 mL distilled water and 3 mL hydrofluoric acid. Microstructural examinations of the AMPs were also carried out using a scanning electron microscope (SEM, FEI, Hillisboro, OR, USA). SEM examination was carried out on the long-transverse sections of the cylindrical friction deposits using secondary electron (SE) imaging modes. Moreover, the grain size of all AM specimens and the base metal have been analyzed by the grain interception method using Olympus Stream Motion Software. A Vickers Hardness Tester (Qness Q10, GmbH, Golling, Austria) with 0.2 kg load and 15 s dwell time was used to evaluate the average hardness of the starting and the AMPs. This test was carried out according to ASTM E92 by measuring twelve readings at least for each AM specimen on the longitudinal sections of the cylindrical friction deposits. The hardness maps were also drawn by collecting four horizontal (perpendicular to building direction) lines and five vertical lines measurements across the AMPs. The free space between any two indentations was 2 mm.

## 3. Results and Discussions

### 3.1. Fabrication of AMPs

For conducting the friction stir deposition and forming the AMPs, the axis of the consumable rod is positioned exactly in the center of the square-shaped substrate to ensure the symmetry and homogeneity of heat dissipation through the substrate. Preliminary tests have been carried out to view the behavior of the rod to avoid buckling, physical discontinuities, or other defects of the rod and ensure the build of the part. Based on these preliminary tests, the rotation speeds, feed rate, and length of the consumable rod have been chosen. In addition, the length of the consumable rod out of the shank holder is varied with the temper condition, as the soft alloy tends to buckle easier than the hard alloy that allows more length to be used.

**Figure 3:**
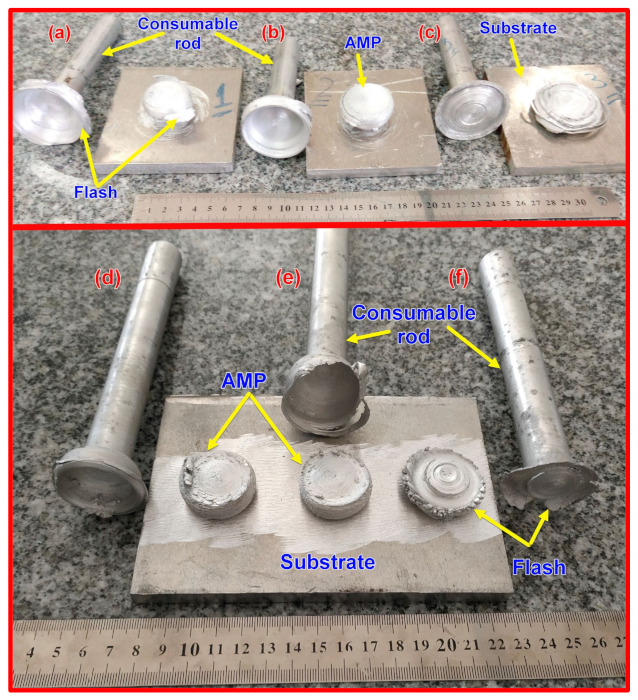
Optical images for the AMPs using FSD and their rods counterparts that remain after obtaining the required part length. AA2011-T6 AMPs processed at 1200 rpm and feeding speeds: (**a**) 3 mm/min, (**b**) 6 mm/min, and (**c**) 9 mm/min. The AA2011-O AMPs processed at 200 rpm and feeding speeds: (**d**) 1 mm/min, (**e**) 2 mm/min, and (**f**) 3 mm/min.

The rubbing between the two surfaces during the rotation and feeding speed of the consumable rod generates frictional heat, which softens the rod’s rubbing end, causing plasticized material at the abutting ends. As the process continues, more plasticized material is built up [14,15]. As the required plasticized material thickness is gained, the rotating consumable rod is stopped and withdrawn; this process promotes a deposited layer on the substrate due to torsional shear. Figure 3 illustrates the remains of AA2011-T6 and AA2011-O consumable rods and their AMPs. Figure 3a–c shows the produced AA2011-T6 AMPs fabricated at a constant rotation rate of 1200 rpm at different feeding speeds of 3, 6, and 9 mm/min, respectively. For the AA2011-O specimens, the consumable rods of AA2011-O are softer than the AA2011-T6 rods. Therefore, the energy required to soften the AA2011-O consumable rod is lower than that needed for softening the AA2011-T6 one [30]. Thus, the AM process was conducted after many trials at a constant rotation rate of 200 rpm and various feeding speeds of 1, 2, and 3 mm/min, as shown in Figure 3d–f, respectively. It should be remarked that the higher feeding speeds of 9 mm/min and 3 mm/min at the rotational rates of 1200 and 200 rpm, respectively, are not recommended to fabricated AMPs of AA2011 alloys, where it is not easy to build continuous multi-layers upward to specific height and diameter. The increase in heat input due to an increase in feeding speed over the optimum condition also produces excessive flash around the AMPs, as given in Figure 3c for AA2011-T6 AMP and Figure 3f for AA2011-O AMP. Thus, it was noted that the conical shape at the end of the consumable rods after finishing the FSD process is flattened in a thin thickness, where the other materials are transferred to flash around the fabricated AMPs.

### 3.2. Macrostructure Examination

**Figure 4:**
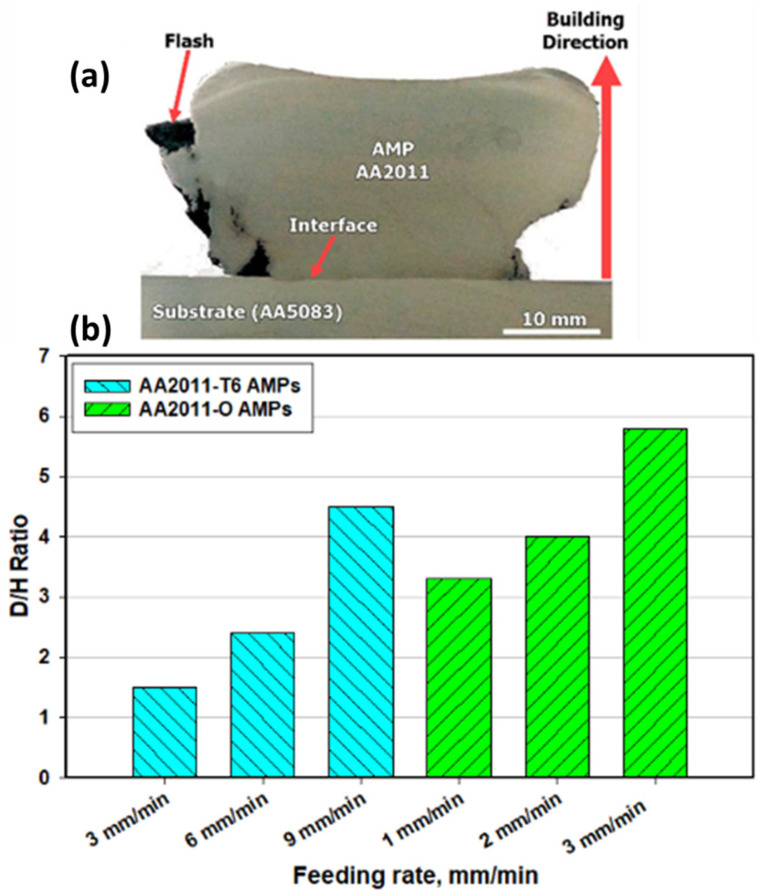
(**a**) The transverse cross-section macrograph for the AMP with the substrate with the building direction and the interface are indicated for the AMP at a constant rotational spindle rate of 1200 rpm and 6 mm/min feeding speeds, (**b**) AMPs Diameters/Height Ratio against feeding speed for all AMPs produced using the different temper conditions and different processing parameters.

Figure 4 illustrates (a) a macrograph of an example of the produced AMP and (b) the AMPs Diameters/Height (D/H) Ratio as a function of the processing feed speed. The visual inspection of the deposit showed that there is significant flash produced from the deposit, which was restacked to the consumable rod. This may be an indication for an overfed condition, in which the feeding speed for the feedstock consumable material is slightly high [16,29]. The possible decrease of the input material feeding speed would mitigate the generation of this produced excess flash. The generation of excessive flash may require post-processing if the geometric accuracy of the final product is sensitive [4]. The macrostructure cross-sections of the produced AMPs show fully continuous dense structures (Figure 3a) without any physical discontinuities or bonding defects at the layer interfaces, indicating the judicious choice of the processing parameters for the AA2011-T6 and AA2011-O aluminum alloys. It can be seen that the D/H of the produced AMPs increases with increasing feeding speed at constant rotation rate for both the AA2011-T6 and the AA2011-O starting materials, as given in Figure 3b. The AA2011 material plasticity during the FSD process is controlled by the amount of heat input introduced in the vortex zone through the AMPs material building from down to up. This phenomenon appears clearly in the AMP geometry based on the applied processing parameters [24].

### 3.3. Microstructure Examination

FSD as a thermomechanical process is similar to friction stir welding (FSW) [29,30,31,32] and processing (FSP) [28] in heat generation, heat dissipation, and heat transfer mechanisms in the stir zone [33,34,35]. In the AA2011 AMPs, the heat is generated by dynamic contact friction (DCF) between the consumable tool and AA5083 substrate material. Then, it causes severe plastic deformation of the AA2011 material under the applied downward force and transfers it to continuous build by material flow during the stir deposition process. FSW and FSP generate localized grain refinement in the whole nugget zone (NZ) behind the rotating pin tool. The FSD material is analogous to the NZ in FSW and FSP [17,36]. It was found that the presence of a refined, equiaxed grain structure engaged with the formation of high-angle grain boundaries is an indication of the dynamic recrystallization in FSW of AA2219-T8 [9] and FSP of AA2024/Al_2_O_3_ nanocomposite [28].

**Figure 5:**
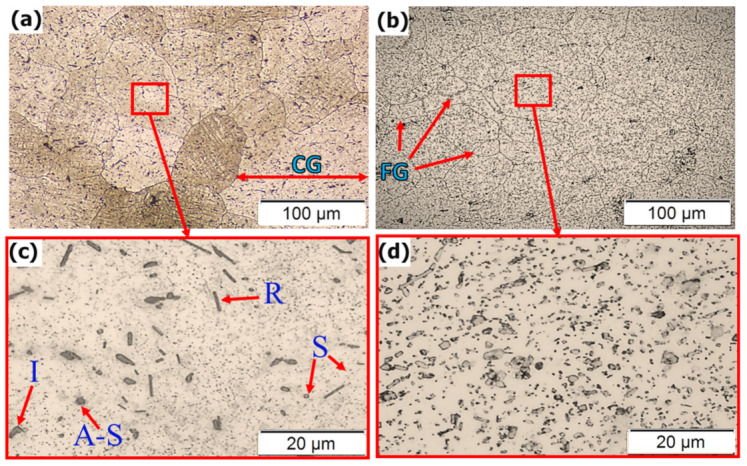
Low and high-magnification optical micrographs of the different temper conditions base material: (**a**,**c**) AA2011-T6 and (**b**,**d**) AA2011-O.

Moreover, Rutherford et al. [9] reported that the reduction in the average volume fraction and size of the intermetallic particles could also be attributed to the severe plastic deformation of the FSD process. Figure 5 shows the optical micrographs of the initial conditions of AA2011 alloys. The microstructure of the AA2011-T6 alloy shows coarse grains as well as the presence of intermetallics in different shapes: rod-like (R), irregular (I), spherical (S), and almost spherical (A-S), as shown in Figure 5a,c. The microstructure grain size in Figure 5c ranges from 30 ± 3 μm to 150 ± 2 μm with an average grain size of 45 ± 8 μm. [37], whereas the microstructure of the AA2011-O rod alloy shows a relatively smaller grain size (Figure 5b,d) than the AA2011-T6 alloy’s grain size. The grain size ranged from 8 ± 2 to 75 ± 3 μm with an average grain size of 16 ± 4 μm. Furthermore, the annealing process causes coarsening of the second phase precipitates compared with the AA2011-T6 material, transferring their shape from the rod-like shape (Figure 5c) to more spheroidal-shaped precipitates (Figure 5d).

#### 3.3.1. AMPs Parts Produced from AA2011-T6 Alloy

**Figure 6:**
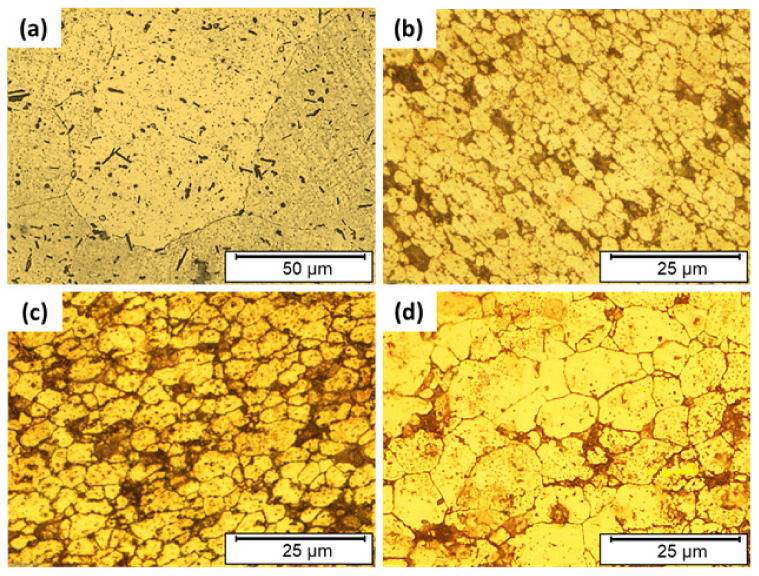
Optical microstructures of the as-received AA2011-T6 (**a**), and AMPs produced t at a rotation rate of 1200 rpm and different feeding speeds of (**b**) 3 mm/min, (**c**) 6 mm/min, and (**d**) 9 mm/min.

The friction-based processes contribute to the increase of temperature of the material in the stirring zone to the temperature range between 60% and 90% of the melting point of the processed material, which is high enough for the recrystallization during the intensive plastic deformation through the solid-state deposition process [38,39]. Figure 6 represents the microstructures of the AA2011-T6 (Figure 6a) and the AMPs deposited at 1200 rpm spindle rotation rate and different feeding speeds of 3 mm/min (Figure 6b), 6 mm/min (Figure 6c), and 9 mm/min (Figure 6d). It can be seen that the coarse grain structure and precipitates of the AA2011-T6 are refined with the applied FSD process parameters at feed speeds of 3, 6, and 9 m/min. The mean measured grain sizes of AA2011-T6 AMPs were 2.9 ± 0.3, 5.3 ± 0.4, and 11.8 ± 0.5 μm at feeding speeds of 3, 6, and 9 mm/min, respectively. It can be said that the reduction in grain size of AMPs deposited at 3, 6, and 9 mm/min feeding speeds reaches the values of 95.3%, 91.5%, and 82.25%, respectively, compared to the grain size of the AA2011-T6 initial material (62 ± 4 μm). The same results of very fine grains and refined second-phase particles are obtained by Dilip et al. [40] for the multi-layer friction deposits of AA2014-T6 (Al–Cu–Mg–Si alloy system). Consequently, the produced AMPs materials undergo continuous dynamic recrystallization and develop very fine equiaxed grains and refined precipitates [3,41]. In addition, Rutherford et al. [9] reported a significant reduction in the intermetallic particles and grain size after FSD processing of AA6061.

#### 3.3.2. AMPs Parts Produced from AA2011-O

**Figure 7:**
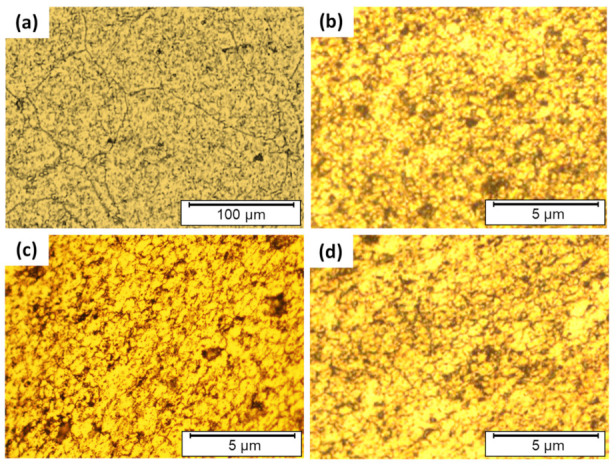
Optical microstructures of the as-received AA2011-O (**a**) and AMPs produced from AA2011-O at a rotation rate of 200 rpm and different feeding speeds of (**b**) 1 mm/min, (**c**) 2 mm/min, and (**d**) 3 mm/min.

Figure 7 represents the microstructures of the AA2011-O (Figure 7a) and the AMPs deposited at 200 rpm spindle rotation rate and different feeding speeds of 1 mm/min (Figure 7b), 2 mm/min (Figure 7c), and 3 mm/min (Figure 7d). A homogenous fine equiaxed structure has been noticed in all conditions due to the stirring of the grains accompanied with dynamic recrystallization during the additive friction-based process [11,20,42]. The mean grain sizes of AA2011-O AMPs were 0.84 ± 0.05, 0.88 ± 0.06, and 0.94 ± 0.08 μm at feeding speeds 1, 2, and 3 mm/min, respectively. It can be reported that the reduction in grain size of AMPs after FSD reaches not less than the value of ≈98% compared to the grain size of the AA2011-O as-received material (48 ± 4 μm) without any significant difference between the applied feeding speeds.

**Figure 8:**
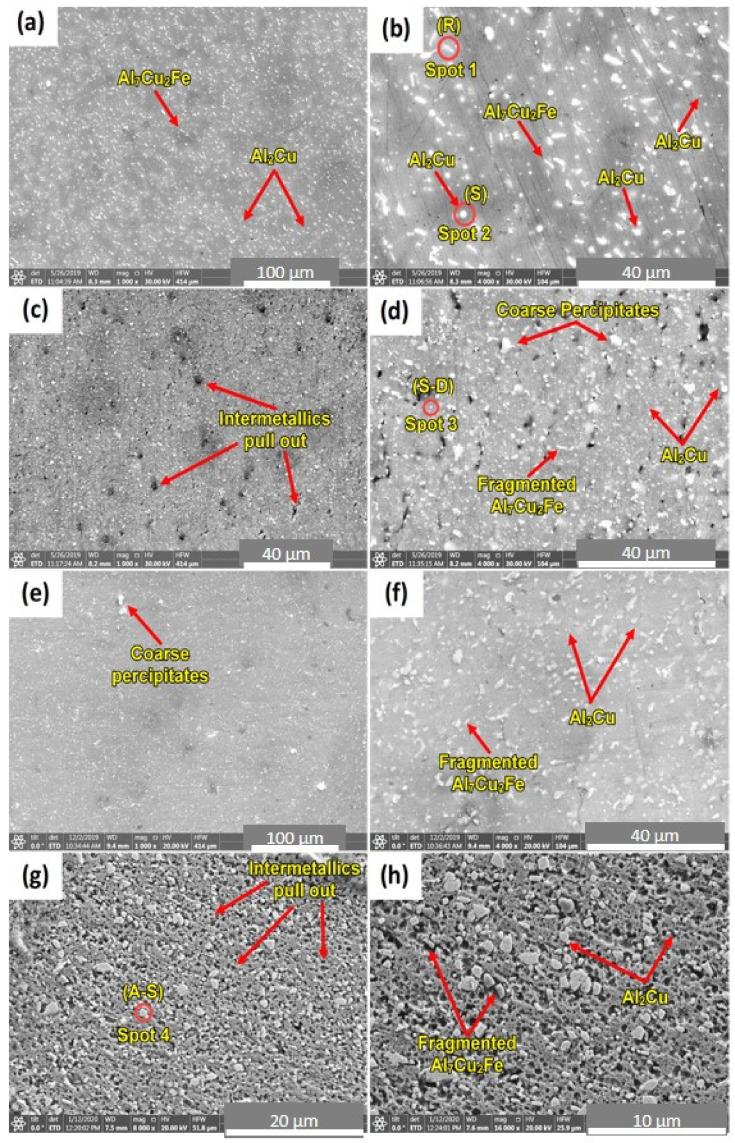
Low and high-magnification SEM micrographs of (**a**,**b**) AA2011-T6 base alloy, (**c**,**d**) FSDed of AA2011-T6 at 1200 rpm—3 mm/min, (**e**,**f**) FSDed AA2011-T6 at 1200 rpm—6 mm/min, and (**g**,**h**) FSDed AA2011-O at 200 rpm—1 mm/min.

SEM was used to examine the present intermetallic precipitates of the as-received AA2011-T6 rod and the friction stir deposited materials at different conditions. Copper is the principal alloying element in AA2011 (Al–Cu alloys). However, other minor alloying elements (Fe, Ti, Zn, and Pb with traces of Ni, Si, and Mn) can also be specified as given in Table 1. During work hardening, an intermetallic phase (Al_2_Cu) is precipitated from a supersaturated solid solution. This intermetallic is crystallographically coherent with the Al matrix. Its fine dispersion improves the hardness and strength of the alloy [43]. The non-deformable second-phase precipitates initially present in the base material AA2011 have been fragmented into a smaller size and got uniformly distributed due to the severe plastic deformation involved in the FSD process; see Figure 8. This fragmentation phenomenon is expected in the stir zone of the friction stir welded materials [28,44] and the friction stir deposited materials [6,10,24].

**Figure 9:**
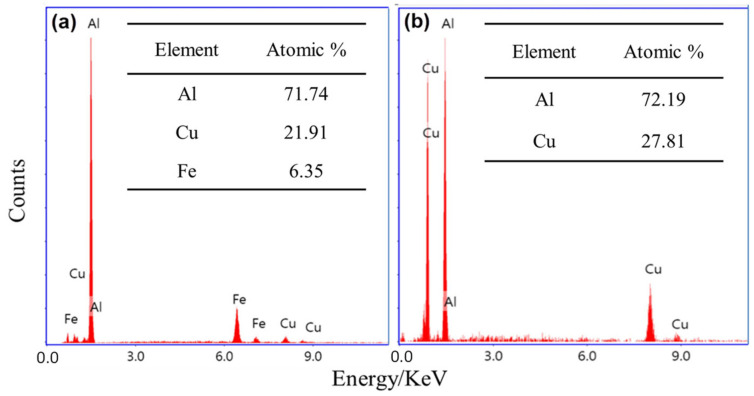
EDS analyses of precipitates show two types of intermetallic precipitates: (**a**) Al_7_Cu_2_Fe (spot 1 analysis in Figure 8) and (**b**) Al_2_Cu (spot 2, 3, and 4 in Figure 8).

The fragmentation of the intermetallics may produce different shapes and sizes. The dispersion of micro and nanoparticles in the aluminum matrix affects the mechanical properties of the AMPs [45,46,47]. Figure 8 shows low and high magnification SEM micrographs of (a) and (b) AA2011-T6 base alloy (c) and (d) FSDed of AA2011-T6 at 1200 rpm–3 mm/min, (e) and (f) FSDed AA2011-T6 at 1200 rpm—6 mm/min, and (g) and (h) FSDed AA2011-O at 200 rpm—1 mm/min. The effect of the stirring process on the fragmentation and distribution of the intermetallic phases can be seen in (Figure 8c–h) compared with the AA2011-T6 base material (Figure 8a,b). Only two types of precipitates were detected in the base material and AMPs; see Figure 9. The EDS analyses of these precipitates are the rod-like shape (R) Al7Cu2Fe (spot 1 analysis in base material; Figure 8b and represented in Figure 9a) and the Al2Cu phase presents in different shapes given the same EDS analyses Figure 9b. These shapes are spherical (S, spot 2 in Figure 8b), small dots (S-D, spot 3 in AMP produced at 1200 rpm and 3 mm/min; Figure 8d), and almost spherical (A-S, spot 4 in AMP produced at 200 rpm and 1 mm/min; Figure 8g). The detected precipitates for both as-received materials and AMPs are consistent with that reported in the literature [37,43]. It can be remarked that the intermetallics are bonded well with the Al matrix for the as-received materials, as shown in Figure 8a,b. Hence, there is no pull-out detected after the grinding and polishing processes. The pull-out of intermetallics is detected for all the AMPs after grinding and polishing (Figure 8c–f). This indicates the weak bond at the interface between the dispersed intermetallics and the Al matrix as a result of subjecting to the FSD thermomechanical process.

**Figure 10:**
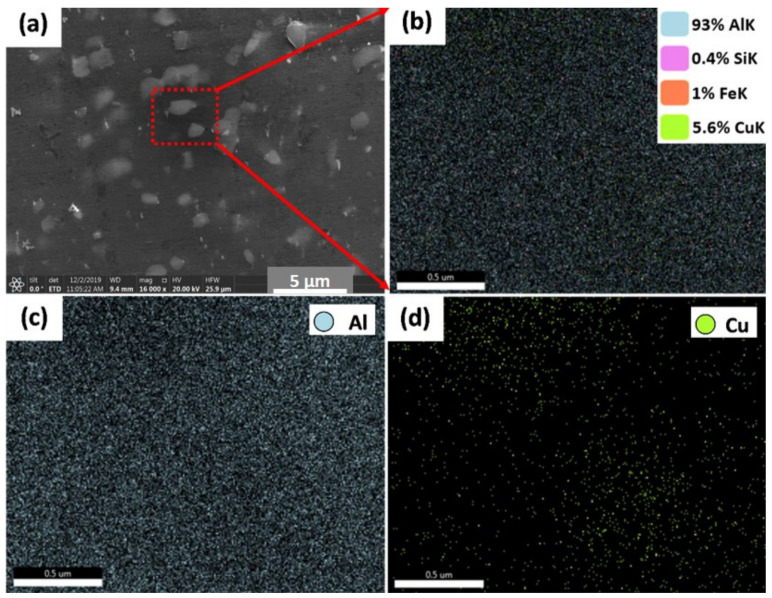
Elemental map distribution of AA2011-T6 AMPs produced at 1200 rpm and 6 mm/min: (**a**) SEM of AMPs, (**b**) map distribution of all alloying elements, and (**c**,**d**) map distribution of Al and Cu, respectively.

**Figure 11:**
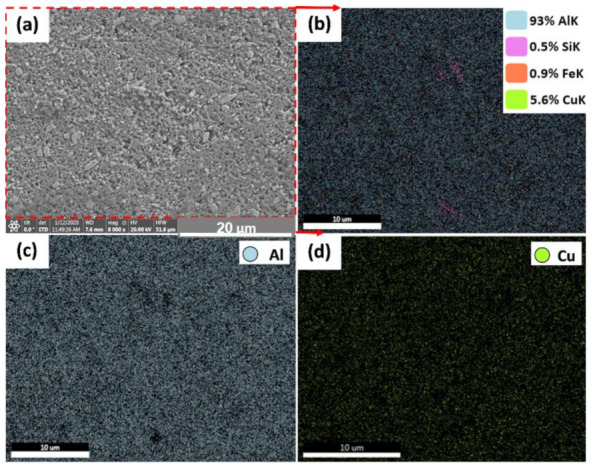
Elemental map distribution of AA2011-O AMPs produced at 200 rpm and 1 mm/min: (**a**) SEM of AMPs, (**b**) map distribution of all alloying elements, and (**c**,**d**) map distribution of Al and Cu, respectively.

Figure 10 and Figure 11 show the EDS elemental mapping of the AMPs produced from AA2011-T6 and AA2011-O at the processing parameters of 1200 rpm spindle rotational rate and 6 mm/min feeding speed and 200 rpm and 1 mm/min, respectively. Figure 10a shows the SEM image obtained from AMP of AA2011-T6 at 1200 rpm and 6 mm/min. It is indicated that the EDS elemental maps confirm the results of the chemical composition of AA2011 in terms of the overall chemical analysis of the alloy as can be seen in Figure 10b. In terms of the elemental maps of the different elements, it can be observed that the Al (c), Fe (e), and Si (f) are homogeneously distributed in their corresponding maps. However, the Cu map in (d) shows a high density of green-colored points where the particles are outlined in the SEM micrographs. Figure 11a shows the SEM image obtained from AMP of AA2011-O at 200 rpm and 1 mm/min. The elemental mapping showed a homogeneous distribution of all elements and phases with no preferences regions along the specimen (no clusters formation), which can be attributed to the severe homogeneous agitation of the material during the FSD process. It is also confirmed the possibility of the Al_7_Cu_2_Fe and Al_2_Cu intermetallics formation and location.

### 3.4. Hardness

**Figure 12:**
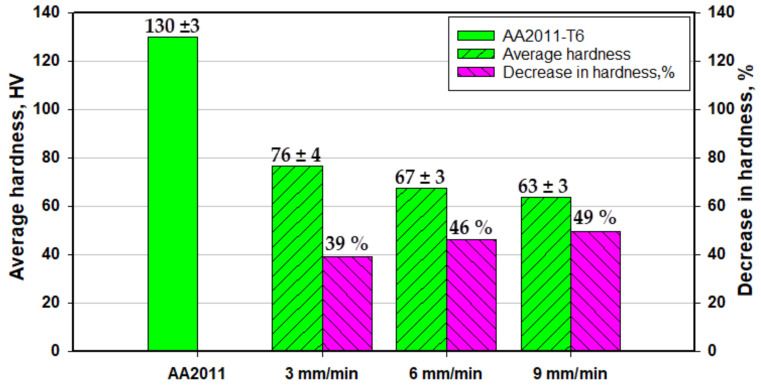
Average hardness and a hardness loss percentage of the AA2011-T6 and its AMPs produced at a rotational rate of 1200 rpm and feed speeds of 3, 6, and 9 mm/min.

**Figure 13:**
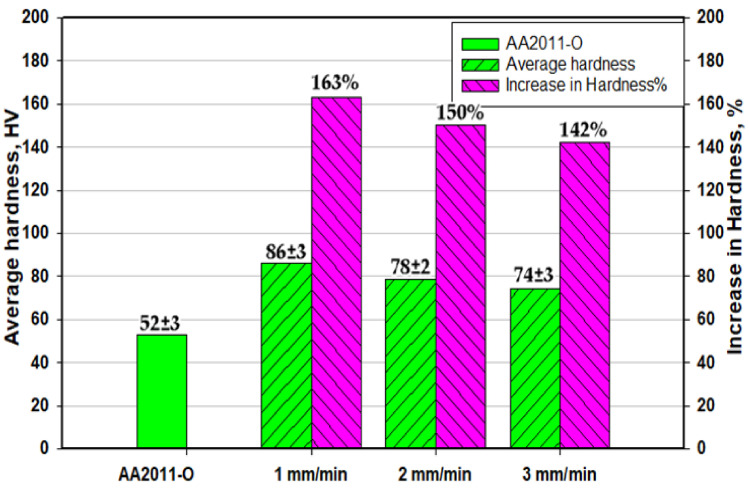
Average hardness and a hardness increase percentage of the AA2011-O and its AMPs produced at a rotational rate of 200 rpm and feed speeds of 1, 2, and 3 mm/min.

Hardness is an important mechanical property to evaluate materials, and its value is controlled by the chemical composition, temper condition, and the FSD process parameters. Figure 12 and Figure 13 represent the average hardness values of the initial materials (AA2011 and AA2011-O) and the produced AMPs at different FSD parameters. The average hardness measurements of the AA2011 AMPs show lower hardness values in comparison to the base metal. Moreover, the hardness decreases as the feeding speed increases, as given in Figure 12. The decrease in hardness percentage (hardness loss %) of AA2011-T6 AMPs reaches the highest value of 49% compared to the AA2011-T6 material at processing parameters of a rotational rate of 1200 rpm and feeding speed of 9 mm/min. At the same rotation rate, the applied feeding speed of 3 mm/min produces an AMP with a hardness loss of 39% compared to the base material. This loss in hardness accompanying the additive manufacturing process was also reported by Dilip et al. [6] for the AMPs of AA2014. They concluded that the friction deposits showed inferior hardness because of their overaged microstructure.

The average hardness values of AA2011-O alloy and the AMPs at a rotation rate of 200 rpm and feeding speeds of 1, 2, and 3 mm/min are illustrated in Figure 13. The hardness value of the AMPs produced at the applied feeding speeds is higher than that of the initial condition AA2011-O. This increase in hardness slightly decreases with the increasing feeding speed. The increase in the hardness value of AMP produced at a 1 mm/min feeding speed attains 163% compared with the AA2011-O starting material, whereas the improvement reaches 142% at the applied feeding speed of 3 mm/min.

The high hardness of the as-received AA2011-T6 (130 ± 3 HV) is ascribed to the high internal stresses stored in the material due to the previous production process, such as cold working. Afterward, the annealing process at 415 °C for 2.5 h followed by slow furnace cooling contributed to the stress relief of the alloy, coarsening, and softening of the second phase precipitates. Thus, the hardness of the alloy decreased after annealing, despite the reduction in grain size. It is well known that two mechanisms are controlling the hardness of this alloy. The first one is the reduction in grain size, which is directly proportional to the hardness increase. The second one is the morphology and dispersion of the second phase precipitates. In AMPs fabricated using the hard rods of AA2011-T6, the hardness decreased after deposition because the dominant mechanism that affects the hardness was the fragmentation of the hard precipitates. On the contrary, the hardness of the AMPs manufactured using the soft AA2011-O rods increased after friction deposition as the dominant mechanism, in this case, was the grain size reduction.

## 4. Conclusions

The current study investigates the effect of alloy temper conditions on the behavior upon friction stir deposition. The deposited AMPs were characterized in terms of macro and microstructural features as well as the hardness distribution. Based on the obtained results, the following conclusions can be outlined:

- The friction stir deposition technique successfully produced sound continuous multi-layered AA2011 AMPs without any physical discontinuities or interfacial defects between layers along the vertical direction.
- The temper condition of the alloy affects the behavior during FSD, and the parameters that worked with the T6 condition does not work with the O condition alloy. The hard condition alloy required high heat input to reach the state of a good deposition, while the soft condition alloy required low heat input to reach the FSD state.
- The optimum condition for FSD of AA2011-T6 was a rotational rate of 1200 rpm at feeding speeds between 3 and 9 mm/min, while the optimum condition for FSD of AA2011-O was a rotational rate of 200 rpm at feeding speeds between 1 and 3 mm/min.
- Microstructural analysis showed that significant grain refining and intermetallic particle fragmentation have been observed in the AMPs from the two temper conditions of AA2011.
- The fine grain formation is mainly dominated by dynamic recrystallization where the average grain size is reduced by decreasing the feeding speed at a constant rotation rate. The use of a lower rotation rate resulted in more refining due to the lower heat input experienced.
- The use of T6 temper alloy has resulted in AMPs with a lower hardness than the starting material that reached about 61% and 51% of the starting material hardness at feeding rates of 3 and 9 mm/min, respectively. However, the use of O temper alloy has resulted in AMPs with higher hardness than the starting material by about 163% hardness enhancement compared to the starting material.

## Figures and Tables

**Table 1:** Chemical composition of AA2011 aluminum alloy (in wt.%).

Cu	Fe	Si	Ti	Zn	Bi	Pb	Ni	Mn	Bal.
5.60	0.72	0.40	0.35	0.30	0.25	0.20	0.02	0.05	Al

**Table 2:** Consumable rod dimensions and FSD processing parameters.

Consumable Rod			FSD Parameters			
**Material**	**Initial Length** **(mm)**	**Rod Diameter** **(mm)**	**Rotation Rate** **(rpm)**	**Feeding Speeds** **(mm/min)**		
AA2011-T6	200	20	1200	3	6	9
AA2011-O	110	20	200	1	2	3

## Data Availability

The data presented in this study are available on request from the corresponding author. The data are not publicly available due to the extremely large size.
